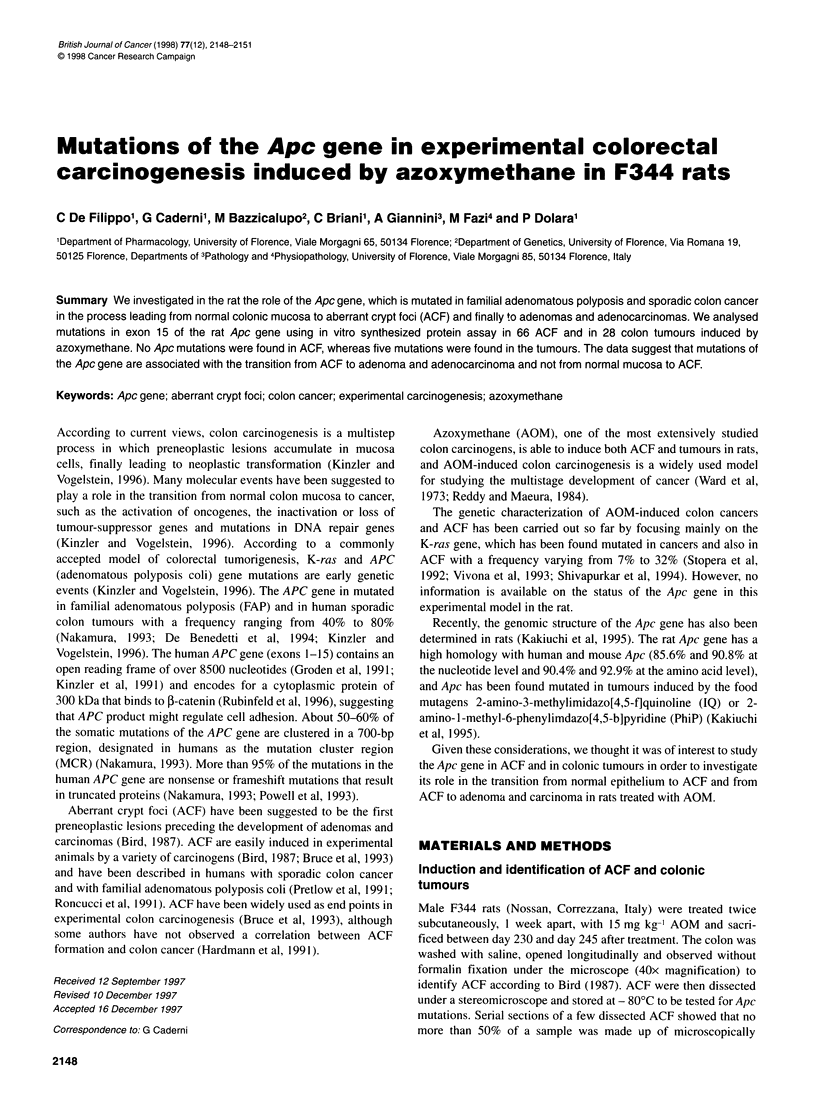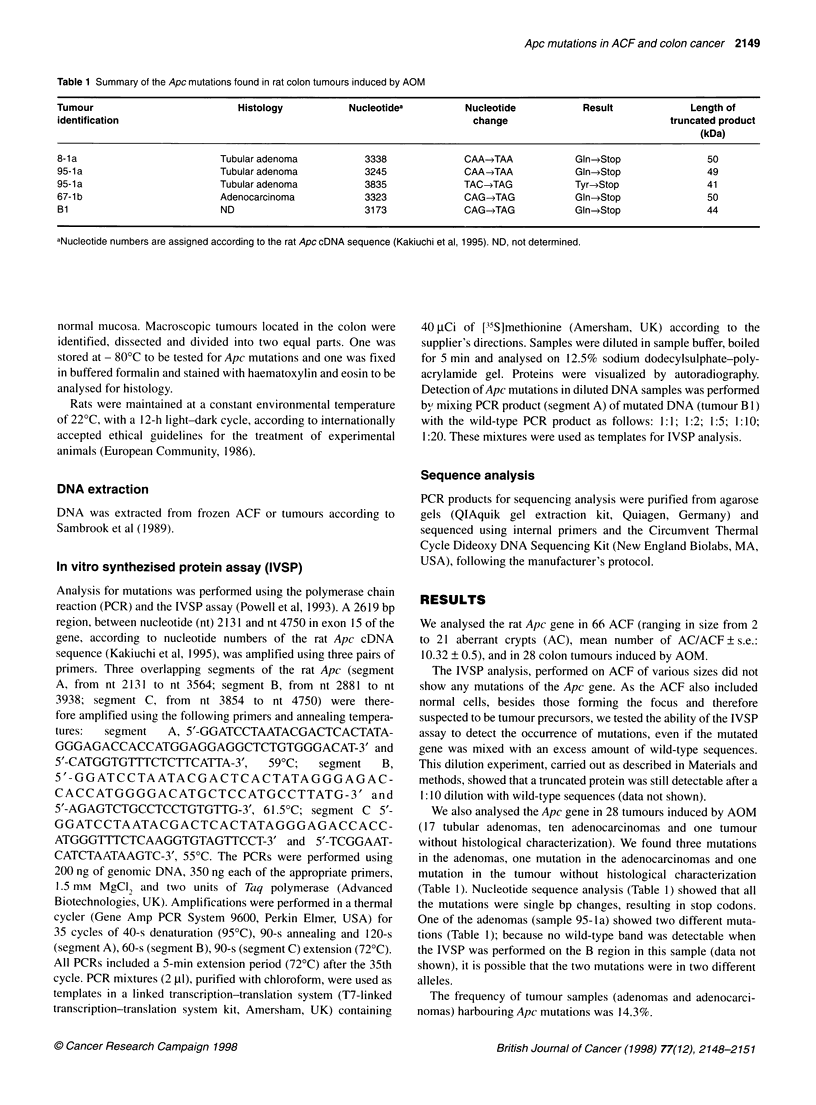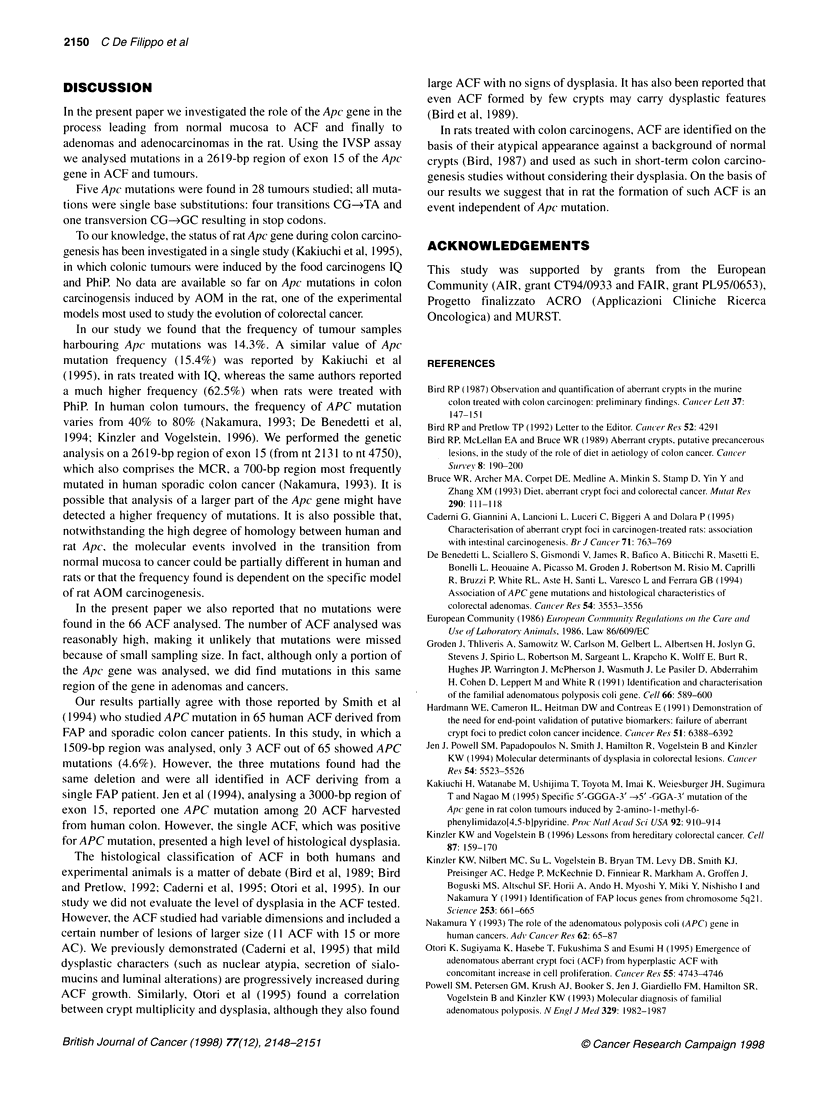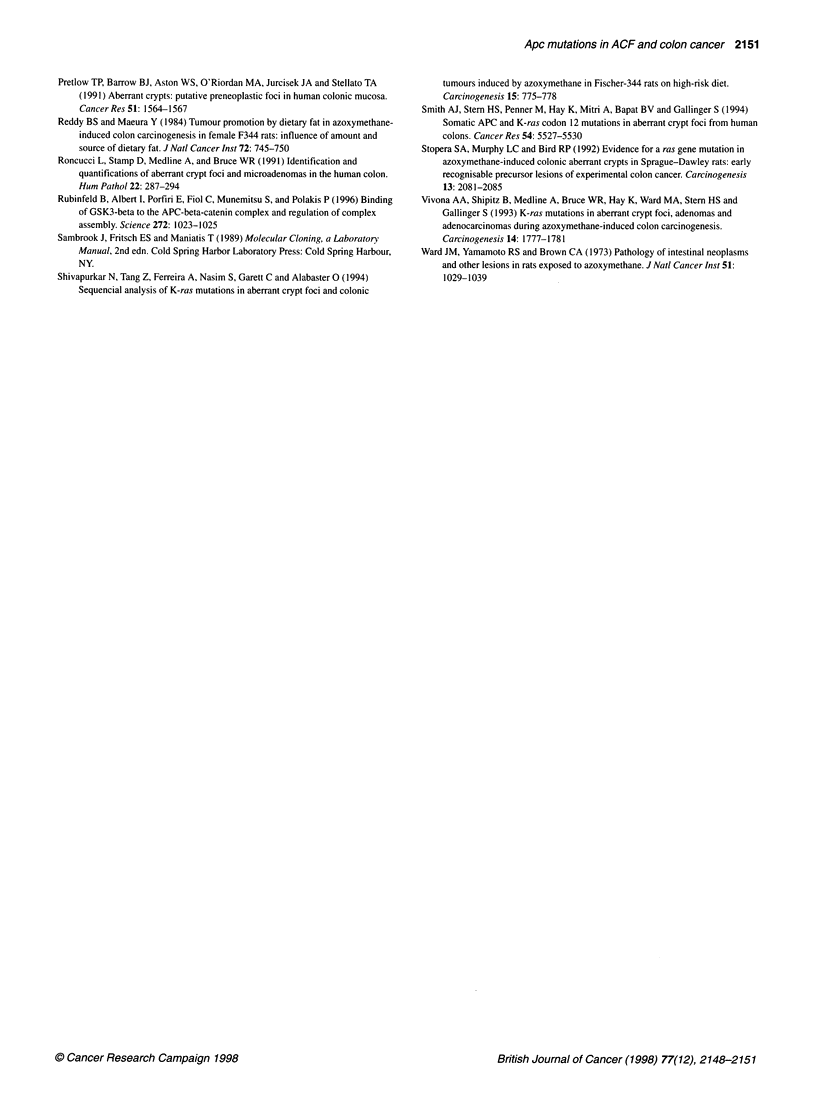# Mutations of the Apc gene in experimental colorectal carcinogenesis induced by azoxymethane in F344 rats.

**DOI:** 10.1038/bjc.1998.359

**Published:** 1998-06

**Authors:** C. De Filippo, G. Caderni, M. Bazzicalupo, C. Briani, A. Giannini, M. Fazi, P. Dolara

**Affiliations:** Department of Pharmacology, University of Florence, Italy.

## Abstract

We investigated in the rat the role of the Apc gene, which is mutated in familial adenomatous polyposis and sporadic colon cancer in the process leading from normal colonic mucosa to aberrant crypt foci (ACF) and finally to adenomas and adenocarcinomas. We analysed mutations in exon 15 of the rat Apc gene using in vitro synthesized protein assay in 66 ACF and in 28 colon tumours induced by azoxymethane. No Apc mutations were found in ACF, whereas five mutations were found in the tumours. The data suggest that mutations of the Apc gene are associated with the transition from ACF to adenoma and adenocarcinoma and not from normal mucosa to ACF.


British Journal of Cancer (1998) 77(12), 2148–2151
© 1998 Cancer Research Campaign

### Mutations of the *Apc* gene in experimental colorectal carcinogenesis induced by azoxymethane in F344 rats

C De Filippo[1], G Caderni[1], M Bazzicalupo[2], C Briani[1], A Giannini[3], M Fazi[4] and P Dolara[1]

[1]Department of Pharmacology, University of Florence, Viale Morgagni 65, 50134 Florence; [2]Department of Genetics, University of Florence, Via Romana 19, 50125 Florence, Departments of [3]Pathology and [4]Physiopathology, University of Florence, Viale Morgagni 85, 50134 Florence, Italy

**Summary** We investigated in the rat the role of the *Apc* gene, which is mutated in familial adenomatous polyposis and sporadic colon cancer in the process leading from normal colonic mucosa to aberrant crypt foci (ACF) and finally to adenomas and adenocarcinomas. We analysed mutations in exon 15 of the rat *Apc* gene using in vitro synthesized protein assay in 66 ACF and in 28 colon tumours induced by azoxymethane. No *Apc* mutations were found in ACF, whereas five mutations were found in the tumours. The data suggest that mutations of the *Apc* gene are associated with the transition from ACF to adenoma and adenocarcinoma and not from normal mucosa to ACF.

**Keywords:** *Apc* gene; aberrant crypt foci; colon cancer; experimental carcinogenesis; azoxymethane

According to current views, colon carcinogenesis is a multistep process in which preneoplastic lesions accumulate in mucosa cells, finally leading to neoplastic transformation (Kinzler and Vogelstein, 1996). Many molecular events have been suggested to play a role in the transition from normal colon mucosa to cancer, such as the activation of oncogenes, the inactivation or loss of tumour-suppressor genes and mutations in DNA repair genes (Kinzler and Vogelstein, 1996). According to a commonly accepted model of colorectal tumorigenesis, K-*ras* and *APC* (adenomatous polyposis coli) gene mutations are early genetic events (Kinzler and Vogelstein, 1996). The *APC* gene in mutated in familial adenomatous polyposis (FAP) and in human sporadic colon tumours with a frequency ranging from 40% to 80% (Nakamura, 1993; De Benedetti et al, 1994; Kinzler and Vogelstein, 1996). The human *APC* gene (exons 1–15) contains an open reading frame of over 8500 nucleotides (Groden et al, 1991; Kinzler et al, 1991) and encodes for a cytoplasmic protein of 300 kDa that binds to β-catenin (Rubinfeld et al, 1996), suggesting that *APC* product might regulate cell adhesion. About 50–60% of the somatic mutations of the *APC* gene are clustered in a 700-bp region, designated in humans as the mutation cluster region (MCR) (Nakamura, 1993). More than 95% of the mutations in the human *APC* gene are nonsense or frameshift mutations that result in truncated proteins (Nakamura, 1993; Powell et al, 1993).

Aberrant crypt foci (ACF) have been suggested to be the first preneoplastic lesions preceding the development of adenomas and carcinomas (Bird, 1987). ACF are easily induced in experimental animals by a variety of carcinogens (Bird, 1987; Bruce et al, 1993) and have been described in humans with sporadic colon cancer and with familial adenomatous polyposis coli (Pretlow et al, 1991; Roncucci et al, 1991). ACF have been widely used as end points in experimental colon carcinogenesis (Bruce et al, 1993), although some authors have not observed a correlation between ACF formation and colon cancer (Hardmann et al, 1991).

*Received 12 September 1997*
*Revised 10 December 1997*
*Accepted 16 December 1997*

*Correspondence to:* G Caderni

Azoxymethane (AOM), one of the most extensively studied colon carcinogens, is able to induce both ACF and tumours in rats, and AOM-induced colon carcinogenesis is a widely used model for studying the multistage development of cancer (Ward et al, 1973; Reddy and Maeura, 1984).

The genetic characterization of AOM-induced colon cancers and ACF has been carried out so far by focusing mainly on the K-*ras* gene, which has been found mutated in cancers and also in ACF with a frequency varying from 7% to 32% (Stopera et al, 1992; Vivona et al, 1993; Shivapurkar et al, 1994). However, no information is available on the status of the *Apc* gene in this experimental model in the rat.

Recently, the genomic structure of the *Apc* gene has also been determined in rats (Kakiuchi et al, 1995). The rat *Apc* gene has a high homology with human and mouse *Apc* (85.6% and 90.8% at the nucleotide level and 90.4% and 92.9% at the amino acid level), and *Apc* has been found mutated in tumours induced by the food mutagens 2-amino-3-methylimidazo[4,5-f]quinoline (IQ) or 2-amino-1-methyl-6-phenylimdazo[4,5-b]pyridine (PhiP) (Kakiuchi et al, 1995).

Given these considerations, we thought it was of interest to study the *Apc* gene in ACF and in colonic tumours in order to investigate its role in the transition from normal epithelium to ACF and from ACF to adenoma and carcinoma in rats treated with AOM.

### MATERIALS AND METHODS

### Induction and identification of ACF and colonic tumours

Male F344 rats (Nossan, Correzzana, Italy) were treated twice subcutaneously, 1 week apart, with 15 mg $kg^{-1}$ AOM and sacrificed between day 230 and day 245 after treatment. The colon was washed with saline, opened longitudinally and observed without formalin fixation under the microscope (40× magnification) to identify ACF according to Bird (1987). ACF were then dissected under a stereomicroscope and stored at – 80°C to be tested for *Apc* mutations. Serial sections of a few dissected ACF showed that no more than 50% of a sample was made up of microscopically

**Table 1** Summary of the *Apc* mutations found in rat colon tumours induced by AOM

| Tumour identification | Histology | Nucleotide[a] | Nucleotide change | Result | Length of truncated product (kDa) |
|---|---|---|---|---|---|
| 8-1a | Tubular adenoma | 3338 | CAA→TAA | Gln→Stop | 50 |
| 95-1a | Tubular adenoma | 3245 | CAA→TAA | Gln→Stop | 49 |
| 95-1a | Tubular adenoma | 3835 | TAC→TAG | Tyr→Stop | 41 |
| 67-1b | Adenocarcinoma | 3323 | CAG→TAG | Gln→Stop | 50 |
| B1 | ND | 3173 | CAG→TAG | Gln→Stop | 44 |

[a]Nucleotide numbers are assigned according to the rat *Apc* cDNA sequence (Kakiuchi et al, 1995). ND, not determined.

normal mucosa. Macroscopic tumours located in the colon were identified, dissected and divided into two equal parts. One was stored at – 80°C to be tested for *Apc* mutations and one was fixed in buffered formalin and stained with haematoxylin and eosin to be analysed for histology.

Rats were maintained at a constant environmental temperature of 22°C, with a 12-h light–dark cycle, according to internationally accepted ethical guidelines for the treatment of experimental animals (European Community, 1986).

### DNA extraction

DNA was extracted from frozen ACF or tumours according to Sambrook et al (1989).

### In vitro synthezised protein assay (IVSP)

Analysis for mutations was performed using the polymerase chain reaction (PCR) and the IVSP assay (Powell et al, 1993). A 2619 bp region, between nucleotide (nt) 2131 and nt 4750 in exon 15 of the gene, according to nucleotide numbers of the rat *Apc* cDNA sequence (Kakiuchi et al, 1995), was amplified using three pairs of primers. Three overlapping segments of the rat *Apc* (segment A, from nt 2131 to nt 3564; segment B, from nt 2881 to nt 3938; segment C, from nt 3854 to nt 4750) were therefore amplified using the following primers and annealing temperatures: segment A, 5′-GGATCCTAATACGACTCACTATA-GGGAGACCACCATGGAGGAGGCTCTGTGGGACAT-3′ and 5′-CATGGTGTTTCTCTTCATTA-3′, 59°C; segment B, 5′-GGATCCTAATACGACTCACTATAGGGAGAC-CACCATGGGGACATGCTCCATGCCTTATG-3′ and 5′-AGAGTCTGCCTCCTGTGTTG-3′, 61.5°C; segment C 5′-GGATCCTAATACGACTCACTATAGGGAGACCACC-ATGGGTTTCTCAAGGTGTAGTTCCT-3′ and 5′-TCGGAAT-CATCTAATAAGTC-3′, 55°C. The PCRs were performed using 200 ng of genomic DNA, 350 ng each of the appropriate primers, 1.5 mM $MgCl_2$ and two units of *Taq* polymerase (Advanced Biotechnologies, UK). Amplifications were performed in a thermal cycler (Gene Amp PCR System 9600, Perkin Elmer, USA) for 35 cycles of 40-s denaturation (95°C), 90-s annealing and 120-s (segment A), 60-s (segment B), 90-s (segment C) extension (72°C). All PCRs included a 5-min extension period (72°C) after the 35th cycle. PCR mixtures (2 µl), purified with chloroform, were used as templates in a linked transcription–translation system (T7-linked transcription–translation system kit, Amersham, UK) containing 40 µCi of [$^{35}$S]methionine (Amersham, UK) according to the supplier's directions. Samples were diluted in sample buffer, boiled for 5 min and analysed on 12.5% sodium dodecylsulphate–polyacrylamide gel. Proteins were visualized by autoradiography. Detection of *Apc* mutations in diluted DNA samples was performed by mixing PCR product (segment A) of mutated DNA (tumour B1) with the wild-type PCR product as follows: 1:1; 1:2; 1:5; 1:10; 1:20. These mixtures were used as templates for IVSP analysis.

### Sequence analysis

PCR products for sequencing analysis were purified from agarose gels (QIAquik gel extraction kit, Quiagen, Germany) and sequenced using internal primers and the Circumvent Thermal Cycle Dideoxy DNA Sequencing Kit (New England Biolabs, MA, USA), following the manufacturer's protocol.

### RESULTS

We analysed the rat *Apc* gene in 66 ACF (ranging in size from 2 to 21 aberrant crypts (AC), mean number of AC/ACF ± s.e.: 10.32 ± 0.5), and in 28 colon tumours induced by AOM.

The IVSP analysis, performed on ACF of various sizes did not show any mutations of the *Apc* gene. As the ACF also included normal cells, besides those forming the focus and therefore suspected to be tumour precursors, we tested the ability of the IVSP assay to detect the occurrence of mutations, even if the mutated gene was mixed with an excess amount of wild-type sequences. This dilution experiment, carried out as described in Materials and methods, showed that a truncated protein was still detectable after a 1:10 dilution with wild-type sequences (data not shown).

We also analysed the *Apc* gene in 28 tumours induced by AOM (17 tubular adenomas, ten adenocarcinomas and one tumour without histological characterization). We found three mutations in the adenomas, one mutation in the adenocarcinomas and one mutation in the tumour without histological characterization (Table 1). Nucleotide sequence analysis (Table 1) showed that all the mutations were single bp changes, resulting in stop codons. One of the adenomas (sample 95-1a) showed two different mutations (Table 1); because no wild-type band was detectable when the IVSP was performed on the B region in this sample (data not shown), it is possible that the two mutations were in two different alleles.

The frequency of tumour samples (adenomas and adenocarcinomas) harbouring *Apc* mutations was 14.3%.

© Cancer Research Campaign 1998

### DISCUSSION

In the present paper we investigated the role of the *Apc* gene in the process leading from normal mucosa to ACF and finally to adenomas and adenocarcinomas in the rat. Using the IVSP assay we analysed mutations in a 2619-bp region of exon 15 of the *Apc* gene in ACF and tumours.

Five *Apc* mutations were found in 28 tumours studied; all mutations were single base substitutions: four transitions CG→TA and one transversion CG→GC resulting in stop codons.

To our knowledge, the status of rat *Apc* gene during colon carcinogenesis has been investigated in a single study (Kakiuchi et al, 1995), in which colonic tumours were induced by the food carcinogens IQ and PhiP. No data are available so far on *Apc* mutations in colon carcinogensis induced by AOM in the rat, one of the experimental models most used to study the evolution of colorectal cancer.

In our study we found that the frequency of tumour samples harbouring *Apc* mutations was 14.3%. A similar value of *Apc* mutation frequency (15.4%) was reported by Kakiuchi et al (1995), in rats treated with IQ, whereas the same authors reported a much higher frequency (62.5%) when rats were treated with PhiP. In human colon tumours, the frequency of *APC* mutation varies from 40% to 80% (Nakamura, 1993; De Benedetti et al, 1994; Kinzler and Vogelstein, 1996). We performed the genetic analysis on a 2619-bp region of exon 15 (from nt 2131 to nt 4750), which also comprises the MCR, a 700-bp region most frequently mutated in human sporadic colon cancer (Nakamura, 1993). It is possible that analysis of a larger part of the *Apc* gene might have detected a higher frequency of mutations. It is also possible that, notwithstanding the high degree of homology between human and rat *Apc*, the molecular events involved in the transition from normal mucosa to cancer could be partially different in human and rats or that the frequency found is dependent on the specific model of rat AOM carcinogenesis.

In the present paper we also reported that no mutations were found in the 66 ACF analysed. The number of ACF analysed was reasonably high, making it unlikely that mutations were missed because of small sampling size. In fact, although only a portion of the *Apc* gene was analysed, we did find mutations in this same region of the gene in adenomas and cancers.

Our results partially agree with those reported by Smith et al (1994) who studied *APC* mutation in 65 human ACF derived from FAP and sporadic colon cancer patients. In this study, in which a 1509-bp region was analysed, only 3 ACF out of 65 showed *APC* mutations (4.6%). However, the three mutations found had the same deletion and were all identified in ACF deriving from a single FAP patient. Jen et al (1994), analysing a 3000-bp region of exon 15, reported one *APC* mutation among 20 ACF harvested from human colon. However, the single ACF, which was positive for *APC* mutation, presented a high level of histological dysplasia.

The histological classification of ACF in both humans and experimental animals is a matter of debate (Bird et al, 1989; Bird and Pretlow, 1992; Caderni et al, 1995; Otori et al, 1995). In our study we did not evaluate the level of dysplasia in the ACF tested. However, the ACF studied had variable dimensions and included a certain number of lesions of larger size (11 ACF with 15 or more AC). We previously demonstrated (Caderni et al, 1995) that mild dysplastic characters (such as nuclear atypia, secretion of sialomucins and luminal alterations) are progressively increased during ACF growth. Similarly, Otori et al (1995) found a correlation between crypt multiplicity and dysplasia, although they also found large ACF with no signs of dysplasia. It has also been reported that even ACF formed by few crypts may carry dysplastic features (Bird et al, 1989).

In rats treated with colon carcinogens, ACF are identified on the basis of their atypical appearance against a background of normal crypts (Bird, 1987) and used as such in short-term colon carcinogenesis studies without considering their dysplasia. On the basis of our results we suggest that in rat the formation of such ACF is an event independent of *Apc* mutation.

### ACKNOWLEDGEMENTS

This study was supported by grants from the European Community (AIR, grant CT94/0933 and FAIR, grant PL95/0653), Progetto finalizzato ACRO (Applicazioni Cliniche Ricerca Oncologica) and MURST.

### REFERENCES

Bird RP (1987) Observation and quantification of aberrant crypts in the murine colon treated with colon carcinogen: preliminary findings. *Cancer Lett* **37**: 147–151

Bird RP and Pretlow TP (1992) Letter to the Editor. *Cancer Res* **52**: 4291

Bird RP, McLellan EA and Bruce WR (1989) Aberrant crypts, putative precancerous lesions, in the study of the role of diet in aetiology of colon cancer. *Cancer Survey* **8**: 190–200

Bruce WR, Archer MA, Corpet DE, Medline A, Minkin S, Stamp D, Yin Y and Zhang XM (1993) Diet, aberrant crypt foci and colorectal cancer. *Mutat Res* **290**: 111–118

Caderni G, Giannini A, Lancioni L, Luceri C, Biggeri A and Dolara P (1995) Characterisation of aberrant crypt foci in carcinogen-treated rats: association with intestinal carcinogenesis. *Br J Cancer* **71**: 763–769

De Benedetti L, Sciallero S, Gismondi V, James R, Bafico A, Biticchi R, Masetti E, Bonelli L, Heouaine A, Picasso M, Groden J, Robertson M, Risio M, Caprilli R, Bruzzi P, White RL, Aste H, Santi L, Varesco L and Ferrara GB (1994) Association of *APC* gene mutations and histological characteristics of colorectal adenomas. *Cancer Res* **54**: 3553–3556

European Community (1986) *European Community Regulations on the Care and Use of Laboratory Animals*, 1986, Law 86/609/EC

Groden J, Thliveris A, Samowitz W, Carlson M, Gelbert L, Albertsen H, Joslyn G, Stevens J, Spirio L, Robertson M, Sargeant L, Krapcho K, Wolff E, Burt R, Hughes JP, Warrington J, McPherson J, Wasmuth J, Le Pasiler D, Abderrahim H, Cohen D, Leppert M and White R (1991) Identification and characterisation of the familial adenomatous polyposis coli gene. *Cell* **66**: 589–600

Hardmann WE, Cameron IL, Heitman DW and Contreas E (1991) Demonstration of the need for end-point validation of putative biomarkers: failure of aberrant crypt foci to predict colon cancer incidence. *Cancer Res* **51**: 6388–6392

Jen J, Powell SM, Papadopoulos N, Smith J, Hamilton R, Vogelstein B and Kinzler KW (1994) Molecular determinants of dysplasia in colorectal lesions. *Cancer Res* **54**: 5523–5526

Kakiuchi H, Watanabe M, Ushijima T, Toyota M, Imai K, Weiesburger JH, Sugimura T and Nagao M (1995) Specific 5′-GGGA-3′ →5′ -GGA-3′ mutation of the *Apc* gene in rat colon tumours induced by 2-amino-1-methyl-6-phenylimidazo[4,5-b]pyridine. *Proc Natl Acad Sci USA* **92**: 910–914

Kinzler KW and Vogelstein B (1996) Lessons from hereditary colorectal cancer. *Cell* **87**: 159–170

Kinzler KW, Nilbert MC, Su L, Vogelstein B, Bryan TM, Levy DB, Smith KJ, Preisinger AC, Hedge P, McKechnie D, Finniear R, Markham A, Groffen J, Boguski MS, Altschul SF, Horii A, Ando H, Myoshi Y, Miki Y, Nishisho I and Nakamura Y (1991) Identification of FAP locus genes from chromosome 5q21. *Science* **253**: 661–665

Nakamura Y (1993) The role of the adenomatous polyposis coli (*APC*) gene in human cancers. *Adv Cancer Res* **62**: 65–87

Otori K, Sugiyama K, Hasebe T, Fukushima S and Esumi H (1995) Emergence of adenomatous aberrant crypt foci (ACF) from hyperplastic ACF with concomitant increase in cell proliferation. *Cancer Res* **55**: 4743–4746

Powell SM, Petersen GM, Krush AJ, Booker S, Jen J, Giardiello FM, Hamilton SR, Vogelstein B and Kinzler KW (1993) Molecular diagnosis of familial adenomatous polyposis. *N Engl J Med* **329**: 1982–1987

*© Cancer Research Campaign 1998*

Pretlow TP, Barrow BJ, Aston WS, O'Riordan MA, Jurcisek JA and Stellato TA (1991) Aberrant crypts: putative preneoplastic foci in human colonic mucosa. *Cancer Res* **51**: 1564–1567

Reddy BS and Maeura Y (1984) Tumour promotion by dietary fat in azoxymethane-induced colon carcinogenesis in female F344 rats: influence of amount and source of dietary fat. *J Natl Cancer Inst* **72**: 745–750

Roncucci L, Stamp D, Medline A, and Bruce WR (1991) Identification and quantifications of aberrant crypt foci and microadenomas in the human colon. *Hum Pathol* **22**: 287–294

Rubinfeld B, Albert I, Porfiri E, Fiol C, Munemitsu S, and Polakis P (1996) Binding of GSK3-beta to the APC-beta-catenin complex and regulation of complex assembly. *Science* **272**: 1023–1025

Sambrook J, Fritsch ES and Maniatis T (1989) *Molecular Cloning, a Laboratory Manual*, 2nd edn. Cold Spring Harbor Laboratory Press: Cold Spring Harbour, NY.

Shivapurkar N, Tang Z, Ferreira A, Nasim S, Garett C and Alabaster O (1994) Sequencial analysis of K-*ras* mutations in aberrant crypt foci and colonic tumours induced by azoxymethane in Fischer-344 rats on high-risk diet. *Carcinogenesis* **15**: 775–778

Smith AJ, Stern HS, Penner M, Hay K, Mitri A, Bapat BV and Gallinger S (1994) Somatic APC and K-*ras* codon 12 mutations in aberrant crypt foci from human colons. *Cancer Res* **54**: 5527–5530

Stopera SA, Murphy LC and Bird RP (1992) Evidence for a *ras* gene mutation in azoxymethane-induced colonic aberrant crypts in Sprague–Dawley rats: early recognisable precursor lesions of experimental colon cancer. *Carcinogenesis* **13**: 2081–2085

Vivona AA, Shipitz B, Medline A, Bruce WR, Hay K, Ward MA, Stern HS and Gallinger S (1993) K-*ras* mutations in aberrant crypt foci, adenomas and adenocarcinomas during azoxymethane-induced colon carcinogenesis. *Carcinogenesis* **14**: 1777–1781

Ward JM, Yamamoto RS and Brown CA (1973) Pathology of intestinal neoplasms and other lesions in rats exposed to azoxymethane. *J Natl Cancer Inst* **51**: 1029–1039

© Cancer Research Campaign 1998